# A novel PID controller for pressure control of artificial ventilator using optimal rule based fuzzy inference system with RCTO algorithm

**DOI:** 10.1038/s41598-023-36506-5

**Published:** 2023-06-07

**Authors:** Debasis Acharya, Dushmanta Kumar Das

**Affiliations:** Department of Electrical and Electronics Engineering, National Institute of Technology Nagaland, Dimapur, Nagaland 797103 India

**Keywords:** Biotechnology, Health care, Engineering, Mathematics and computing

## Abstract

In order to improve the pressure tracking response of an artificial ventilator system, a novel proportional integral derivative (PID) controller is designed in the present work by utilizing an optimal rule-based fuzzy inference system (FIS) with a reshaped class-topper optimization algorithm (RCTO), which is named as (Fuzzy-PID). Firstly, a patient-hose blower-driven artificial ventilator model is considered, and the transfer function model is established. The ventilator is assumed to operate in pressure control mode. Then, a fuzzy-PID control structure is formulated such that the error and change in error between the desired airway pressure and actual airway pressure of the ventilator are set as inputs to the FIS. The gains of the PID controller (proportional gain, derivative gain, and integral gain) are set as outputs of the FIS. A reshaped class topper optimization algorithm (RCTO) is developed to optimize rules of the FIS to establish optimal coordination among the input and output variables of the FIS. Finally, the optimized Fuzzy-PID controller is examined for the ventilator under different scenarios such as parametric uncertainties, external disturbances, sensor noise, and a time-varying breathing pattern. In addition, the stability analysis of the system is carried out using the Nyquist stability method, and the sensitivity of the optimal Fuzzy-PID is examined for different blower parameters. The simulation results showed satisfactory results in terms of peak time, overshoot, and settling time for all cases, which were also compared with existing results. It is observed in the simulation results that the overshoot in the pressure profile is improved by 16% with the proposed optimal rule based fuzzy-PID as compared with randomly selected rules for the system. Settling time and peak time are also improved 60–80% compared to the existing method. The control signal generated by the proposed controller is also improved in magnitude by 80–90% compared to the existing method. With a lower magnitude, the control signal can also avoid actuator saturation problems.

## Introduction

An artificial ventilator is an emergency life-saving medical device used in the intensive care unit (ICU) or anaesthesia workstation for critical patients with breathing problems^1^. It creates enough pressure to allow air flow into the lungs during inspiration and releases pressure to blow out air from the lungs during expiration^1,2^. During serious medical conditions, it aids the patient in reducing their breathing workload^3^. A large number of patients required ventilation during the COVID-19 pandemic that started in 2019^2^. The demands for such medical devices are increasing day by day. The improvement in performance of the artificial ventilator is required for the patient’s safety and comfort during ventilation time.

To help a patient, a ventilator can work in a variety of modes, including regulated mode, aided mode, and spontaneous breathing mode^4^. The ventilator has some control variables, such as constant pressure, volume, or both. For patients in severe conditions or during surgery, a volume-controlled ventilator (VCV) is typically advised. Depending on the resistance and compliance of the respiratory tract, the pressure in the lung may change in VCV, and the danger of barotrauma is significant. However, a pressure controlled ventilator (PCV) used in an intensive care unit (ICU) will support a patient based on a predetermined, steady pressure and can reduce the danger of barotrauma^5^.

Patient-ventilator dyssynchrony is a critical issue with a ventilator^6^. When a patient is receiving artificial ventilation, there is a mismatch between the timing of breathing, air flow, volume, or air pressure, and the capacity of the ventilator to supply those needs. Patient-ventilator dyssynchrony happens when the beginning and ending times of artificial breathing don’t coincide with the patient’s beginning and ending timings for inspiration, respectively. Patient-ventilator dyssynchrony can lead to a variety of adverse effects, including irritability in the patient, alveolar over-distention and lung injury, sleep issues, erratic breathing, unnecessary sedation, excessive demand on the diaphragm, and so on^7^. In order to preserve the proper response from the ventilator, they need a precise and effective control mechanism. It is necessary to synchronise a ventilator’s response with a patient’s breathing pattern when they are receiving ventilation.

**Figure 1:**
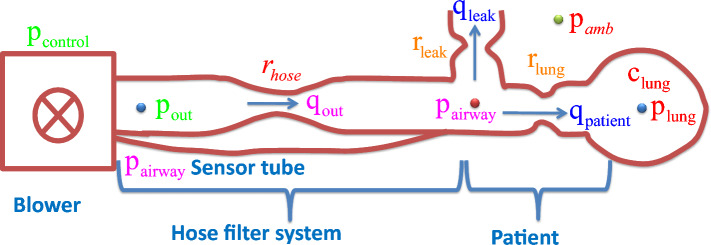
Patient hose-blower driven ventilator model.

A blower-driven patient-hose type pressure-controlled ventilator (PCV) model is generally used in the ICU for sedated patients (Fig. 1). In PCV, the ventilator is required to monitor a desired pressure near the mouth of the patient. The inspiratory peak pressure (IPP) and positive end expiratory pressure (PEEP) allow air to enter and exit the respiratory system, respectively. There are a number of control schemes found in the literature to enhance the pressure monitoring performance of ventilation system^1,8^. In^1^, authors have proposed variable gain control logic for the ventilator model to ensure a fast rise time in the pressure tracking profile with a small overshoot in air flow. In^8^, authors have presented a mathematical modelling and control scheme for artificial ventilators. In^9^, a funnel control system with limited improvement in pressure profile is presented for the ventilator model. In^10,11^, the model predictive strategy is used to design a suitable controller for the ventilation model. In^12^, a learning strategy based iterative control method is presented. For artificial ventilators, an adaptive control is proposed in^13^.

Generally, a PID controller is used in industry as a control mechanism for artificial ventilation system due to its simple structure and ease of implementation. It brings together the benefits of different control actions, such as a faster response time due to the proportional gain, offset-free due to the combination effect of proportional and integral control actions, and disturbance elimination by measuring the change in error (for derivative control actions). Therefore, selecting a PID control structure for the ventilator is justified. There are many analytical methods available to tune such a controller for any system, but they are complex and time-consuming processes where operating scenarios may vary. Trial-and-error methods may also be used, but they will not provide optimal settings for such a controller. Due to this, many authors have developed and recommended swarm optimization algorithms to tune such controllers for optimal system^14^. There are many algorithms available that are successfully applied to tune PID controllers for different engineering control problems, such as particle swarm optimization (PSO)^14,15^, class topper optimization (CTO)^16^, gravitational search algorithm (GSA)^17^ and so on. Among available algorithms, CTO has the advantage of a clustering nature, which allows it to divide the search space into different sections (local search space). Finding the optimal solution among all sections is easy. Therefore, CTO is considered in this paper. Moreover, a suitable balance can effectively be made between exploration and exploitation during the search for an optimal solution. Further, the convergence behaviour of the CTO is upgraded by using the concept of crossover during the updating of solutions by the algorithm. Therefore, CTO can be used to tune the PID controller for the ventilator system. There is an issue of accuracy in a PID controller for a system if the parameters of the controller are optimized with such algorithms. An optimal PID controller with a swarm based technique is a fixed gain PID controller that is optimized for a fixed operating scenario. With the change in operating scenario, such a controller may not perform accurately. They may need to run again and again for a system under different operating scenarios. As the physiological parameters of the lung system vary for different patients, a fixed-gain PID controller may not work efficiently for such a system. Moreover, to design a fixed-gain PID controller for a system, accurate modeling of the lung system is also required. Modeling a physiological system is also a challenging task. In this regard, a fuzzy system can be used as a decision making system that will set the parameters of the PID controller for a system under any disturbance. A fuzzy system can update its parameters on each control cycle. Moreover, they are insensitive to the structure of a system. It can manage a variety of disturbances. It can also be used along with a PID controller to tune the controller under changing environmental or operating conditions, parametric uncertainties, or external disturbances. Therefore, a Fuzzy-PID structure can be justified for the ventilator system. Different applications of fuzzy controllers are found in the literature^18–20^. In^21^, the authors have presented a FLC-based ventilator with 96 different rules based on factors such as heart rate, tidal volume, and breathing rate. In^22^, an internal model control using fuzzy controllers is provided for the perfusion surgery system. In^23^, the author has created a fuzzy controller for ventilators, that includes a fuzzy-based parameter estimate assistance system. In^24^, for a nonlinear respiration model, the authors proposed a fuzzy-neural methodology, in which the fuzzy approach is utilised for flow control and the neural network is employed for breathing mechanics. In^25^, the authors have presented intelligent tuning methods for optimal setting of fuzzy logic controller. In^26^, authors have designed an optimal rule based fuzzy system with an improved genetic algorithm to control throttle valves for managed pressure drilling (MPD) systems. In^27^, a fuzzy system is developed for electromechanical devices to predict and classify faults. In^28^, a hybrid fuzzy expert system is proposed as a decision-support tool to lessen the risk linked with petrol gearbox stations.

The fundamental problem with fuzzy control systems is that they require specialised expertise to operate properly for a given system. The expert knowledge base rules must work well together with the MFs of fuzzy inputs and outputs. Sometimes, it is difficult to get expert-knowledge-based rules for a system to make an efficient fuzzy controller. The mismatch of opinions among different experts about a system’s response may lead to the selection of improper rules for a fuzzy system. Therefore, a fuzzy-PID control structure is proposed in this paper, with the outputs of the fuzzy inference system (FIS) being the gains of the PID controller (proportional gain, derivative gain, and integral gain) and the inputs of the FIS being the error and change in error between the desired airway pressure and actual airway pressure of the ventilator. In order to establish the best coordination between the input and output variables of the FIS, a reshaped class topper optimisation algorithm (RCTO) is used to decide optimal rule base for the systems. Under a variety of conditions, including parametric uncertainties, outside disturbances, sensor noise, and a time-varying breathing pattern, the optimised Fuzzy-PID controller is tested for the ventilator. Additionally, the system’s stability is analysed using the Nyquist stability method, and the sensitivity of the ideal Fuzzy-PID is looked at in relation to various blower parameters.

The rest of the present work is organized as follows: The development of the ventilator mathematical model is shown in the "Mathematical modeling of ventilation system" section. In "Problem formulation" section, the problem formulation of the present work is stated. In "Proposed method of optimizing Fuzzy rules" section, proposed method of optimizing the rules of FIS is discussed in detail. The RCTO algorithm is presented in "Reshaped class topper optimization algorithm-RCTO" section. In "Simulation and result analysis" section, the simulation and result analysis are described. The conclusion and future work are stated in "Conclusion and future work" section.

## Mathematical modeling of ventilation system

In this section, the mathematical representation of the mechanical ventilation system is presented. The working principle of a blower driven patient-hose type ventilator is illustrated in Fig. 1. As shown in Fig. 1, there are three sections in the ventilator, which include the blower, hose, and the lung system of patients. An actuator (electrical motor) is used for the blower system to produce the patient’s required pressure. A hose system is used to link the blower with the patient’s lungs. All symbols and related parameters of the system dynamic are given in Table 1.

**Table 1:** Examples of possible model parameters for simulation.

Symbols	Values
$r_{lungs}$	5 mbar s/l
$c_{lungs}$	20 ml/mbar
$r_{leak}$	60 mbar s/l
$c_{hose}$	4.5 mbar s/l

The airflow from the blower system runs through the hose system towards the patient’s lungs. The blower system causes air-flow at a rate of ${q_{out}}$ towards the lung system of the patient through the hose. Due to the effect of hose system resistance, the flow enters the lungs at a flow rate of ${q_{patient}}$ as shown in Fig. 1. For the exhalation process of patients, a leak on the hose system near the mouth with the leak resistance ${r_{leak}}$ is used such that patients can release ${{\text{CO}}_2}$ air at a rate ${q_{leak}}$. Therefore, the air-flow dynamics can be expressed as follows^29^:

1 $$\begin{aligned} {q_{patient}}={q_{out}}-{q_{leak}}, \end{aligned}$$

From the diagram in Fig. 1, one can derive the following expressions for the flow rate of the blower, lungs, and leak considering nominal hose resistance (${r_{leak}}$), leak (${r_{leak}}$) and lungs system resistance (${r_{lungs}}$) as follows:

2 $$\begin{aligned} {q_{out}}= & {} \frac{{p_{out}}-{p_{airway}}}{r_{hose}}, \end{aligned}$$

3 $$\begin{aligned} {q_{leak}}= & {} \frac{{p_{airway}}}{{r_{leak}}}, \end{aligned}$$

4 $$\begin{aligned} {q_{patient}}= & {} \frac{{p_{airway}}-{p_{lungs}}}{{r_{lungs}}}. \end{aligned}$$

Using (2) to (4) into (1) and rearranging, one obtains

5 $$\begin{aligned} {{p_{airway}}}=\frac{\frac{1}{{{r}_{lungs}}}{{p}_{lungs}}+\frac{1}{{{r}_{hose}}}{{p}_{out}}}{\frac{1}{{{r}_{hose}}}+\frac{1}{{{r}_{lungs}}}+\frac{1}{{{r}_{leak}}}}. \end{aligned}$$

The dynamic ${p_{lungs}}$ can be defined as follows:

6 $$\begin{aligned} {\dot{p}}_{lungs}=\frac{1}{{c_{lungs}}}\times {q_{patient}}, \end{aligned}$$

Using (4) into (6), one can obtain

7 $$\begin{aligned} {\dot{p}}_{lungs}=\frac{{p_{airway}}-{p_{lungs}}}{{r_{lungs}}{c_{lungs}}}, \end{aligned}$$

(7) is analogous to a conventional *RC* electrical circuit where, ${p_{lungs}}$ is similar to voltage, ${q_{patient}}$ is current caused an air-pressure ${p_{airway}}$ into the lungs at a time constant ${{r_{lungs}}{c_{lungs}}}$.

Using (5) and (7), one gets

8 $$\begin{aligned} {\dot{p}}_{lungs}=\frac{-\left( \frac{1}{{{r}_{hose}}}+\frac{1}{{{r}_{leak}}} \right) {{p}_{lungs}}+\frac{1}{{{r}_{hose}}}{{p}_{0}}}{{{r}_{lungs}}{{c}_{lungs}}\left( \frac{1}{{{r}_{hose}}}+\frac{1}{{{r}_{lungs}}}+\frac{1}{{{r}_{leak}}} \right) }. \end{aligned}$$

Considering ${{p}_{out}}$ as input, ${p_{airway}}$ and ${q_{patient}}$ as outputs of hose-patient ventilation system with ${{p}_{lungs}}$ as a state of the system, using (2), (3), (4), (5), and (8), one can write the system dynamics in state space form as follows:

9 $$\begin{aligned} \nonumber {{\dot{p}}_{lungs}}= & {} {{A}_{hs}}{{p}_{lungs}}+{{B}_{hose}}{{{p}_{out}}}, \\ \left[ \begin{matrix} {{p}_{a}} \\ {{q}_{p}} \\ \end{matrix} \right]= & {} {{C}_{hose}}{{p}_{lungs}}+{{D}_{hose}}{{{p}_{out}}}, \end{aligned}$$

where

$$\begin{aligned} {{A}_{hose}}= & {} {}-\frac{\frac{1}{r_{hose}}+\frac{1}{r_{leak}}}{{{r}_{lungs}}{{c}_{lungs}}\left( \frac{1}{r_{hose}}+\frac{1}{{{r}_{lungs}}}+\frac{1}{r_{leak}} \right) }, \\ {{B}_{hose}}= & {} {}-\frac{\frac{1}{r_{hose}}}{{{r}_{lungs}}{{c}_{lungs}}\left( \frac{1}{r_{hose}}+\frac{1}{{{r}_{lungs}}}+\frac{1}{r_{leak}} \right) }, \\ {{C}_{hose}}= & {} \left[ \begin{array}{ll} \frac{\frac{1}{{{r}_{lungs}}}}{\left( \frac{1}{r_{hose}}+\frac{1}{{{r}_{lungs}}}+\frac{1}{r_{leak}} \right) } &{} -\frac{\frac{1}{r_{hose}}+\frac{1}{r_{leak}}}{{{r}_{lungs}}\left( \frac{1}{r_{hose}}+\frac{1}{{{r}_{lungs}}}+\frac{1}{r_{leak}} \right) }, \\ \end{array} \right] \\ {{D}_{hs}}= & {} \left[ \begin{array}{ll} \frac{\frac{1}{{{r}_{hoss}}}}{\left( \frac{1}{r_{hose}}+\frac{1}{r_{lungs}}+\frac{1}{r_{leak}} \right) } &{} \frac{\frac{1}{r_{hose}}}{{{r}_{lungs}}\left( \frac{1}{r_{hose}}+\frac{1}{{{r}_{lungs}}}+\frac{1}{r_{leak}} \right) }, \\ \end{array} \right] \end{aligned}$$

(9) can be written in terms of transfer function as follows:

10 $$\begin{aligned} G\left( s \right) ={{C}_{hose}}{{\left( sI-{{A}_{hose}} \right) }^{-1}}{{B}_{hose}}+{{D}_{hose}}. \end{aligned}$$

For the inertia, blower system is associated with high frequency. It can be modeled in transfer function form as follows^29^:

11 $$\begin{aligned} {{G}_{blower}}\left( s \right) =\frac{{{p}_{out}}\left( s \right) }{{{p}_{control}}\left( s \right) }=\frac{\omega _{n}^{2}}{{{s}^{2}}+2\varsigma {{\omega }_{n}}s+\omega _{n}^{2}}, \end{aligned}$$

where $${{\omega }_{n}=2{\pi }30}$$ and $${{\varsigma }=1}$$.

## Problem formulation

For the patient hose blower driven ventilator system in (9), an optimal rule base control technique will be created to enhance the ventilator’s capability to monitor airway pressure in the lung system of a critical patient during artificial ventilation.

The mathematical model of PID controller is presented as follows.

12 $$\begin{aligned} C(s) ={{k}_{p}}+{\frac{{{k}_{i}}}{s}}+{{k}_{d}}{s}, \end{aligned}$$

where ${{k}_{p}}$ is the proportional gain, ${{k}_{i}}$ is the integral gain and ${{k}_{d}}$ is the derivative gain.

**Figure 2:**
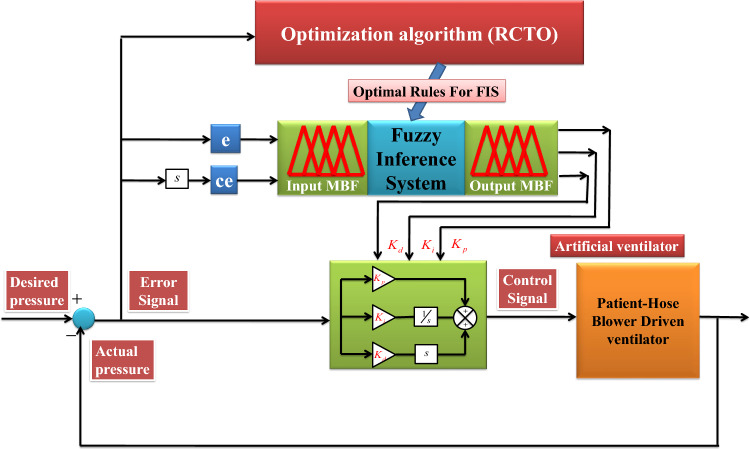
Optimal rule base Fuzzy-PID control scheme for Artificial Ventilator.

The robustness of a PID controller depends on the parameters of the controller under the operating conditions of the system. The PID controller may need to be tuned as the operating conditions of a system change. As a conventional PID controller is not able to self-tune the parameters, the performance of the PID controller will be reduced. Therefore, the concept of fuzzy control logic is used to make the PID controller auto-tune under parametric uncertainties or changes in the operating conditions of the system. A fuzzy controller works efficiently if the coordination between input and output variables of a fuzzy inference system is done accurately. As the FIS of the proposed controller has two inputs (error and change in error of actual airway pressure and desired airway pressure) and three outputs (parameters of the PID controller), there will be 49 rules for each output variable with 7 types of membership functions. Thus, a total of ${49\times 3}$ rules (49 rules for each of proportional, derivative, and integral gains) is required to optimize. For such a large number of optimization variables, a metaheuristic algorithm will be justified to avoid the calculation complexity of analytical methods. For this purpose, a classical algorithm called class topper optimization (CTO) is reshaped to form a reshaped class topper optimizer (RCTO) by utilising the concept of crossover in the updation stage of the classical CTO. During any disturbance or parametric uncertainty in the ventilation system, the gains of the PID controller will be adjusted automatically by the optimal rule-base fuzzy controller to achieve better control action by the PID controller for the ventilator system. A graphical representation of the proposed control scheme is shown in Fig. 2. The detailed procedures are described in the next section.

## Proposed method of optimizing Fuzzy rules

**Step 1: Formulation of Fuzzy Inference System (FIS)**

A rule base fuzzy inference system (FIS) is first created. The steps of designing FIS are as follows:

**Inputs of FIS** Error (*e*) and change in error (*ce*) between desired and actual airway pressure of ventilator are taken as inputs of the FIS. The membership functions (MFs) for both of input variable of FIS are taken as follows:

13 $$\begin{aligned} {MF}_{inputs}=\left[ \text {NL, NM, NS, ZE, PS, PM, PL} \right] , \end{aligned}$$

where NL is negative large, NM is negative medium, NS is negative small, ZE is zero, PS is positive small, PM is positive medium, PB is positive big.

**Figure 3:**
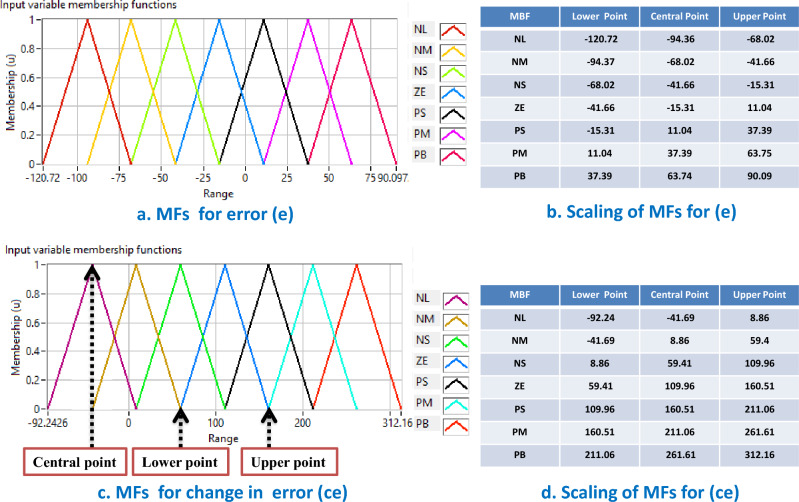
Membership function (MF) of inputs for fuzzy inference system. (**a**) Range, scaling and shape of MFs for error (e). (**b**) Upper, central and lower point of MFs for error (e). (**c**) Range, scaling and shape of MFs for change in error (ce). (**d**) Upper, central and lower point of MFs for change in error (ce)^30^.

In this paper, the setting of MFs for each input variable of the FIS system is taken from^16^. The detailed procedure for obtaining these optimal settings (ranges and different points of MFs) is presented in^16^. A graphical representation of such membership functions with the range and scaling of MFs for inputs to the FIS is presented in Fig. 3.

**Outputs of FIS:** The parameters of PID controller (${{k}_{p}}$, ${{k}_{i}}$ and ${{k}_{d}}$) are taken as outputs of FIS. The membership functions for each output variables of FIS are chosen as follows:

14 $$\begin{aligned} {MF}_{outputs}=\left[ \text {NL, NM, NS, ZE, PS, PM, PL} \right] , \end{aligned}$$

**Figure 4:**
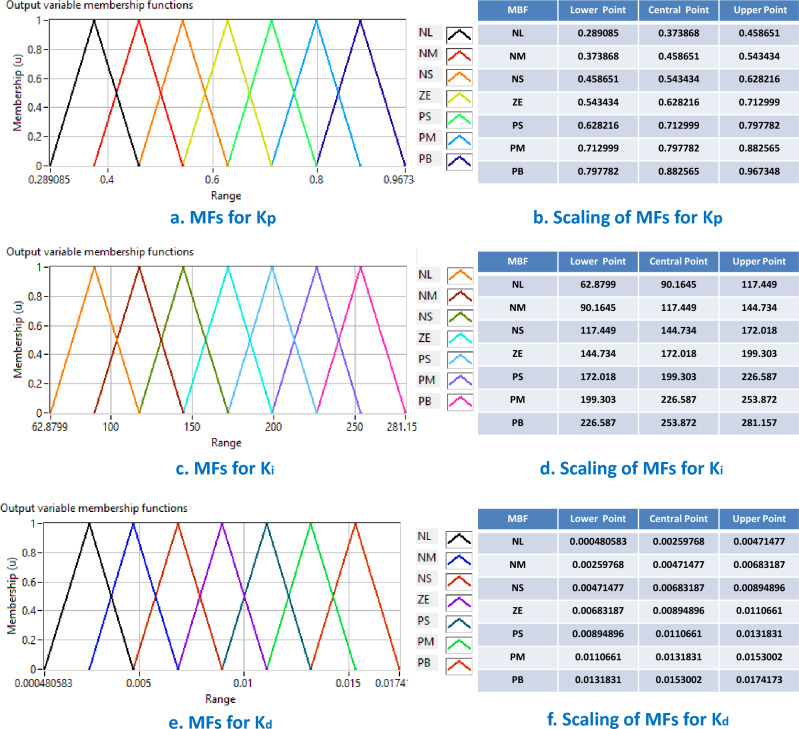
Membership function (MF) of outputs for fuzzy inference system. (**a**) Range, scaling and shape of MFs for ${{k}_{p}}$. (**b**) Upper, central and lower point of MFs for ${{k}_{p}}$. (**c**) Range, scaling and shape of MFs for ${{k}_{i}}$. d. Upper, central and lower point of MFs for ${{k}_{i}}$. (**e**) Range, scaling and shape of MFs for ${{k}_{d}}$. (**f**) Upper, central and lower point of MFs for ${{k}_{d}}$^30^.

The ranges, scaling and shapes of membership functions for fuzzy output variables (${{k}_{p}}$, ${{k}_{i}}$ and ${{k}_{d}}$) of the FIS are taken from^16^. A graphical representation of such membership functions for fuzzy system outputs is presented in Fig. 4.

**Figure 5:**
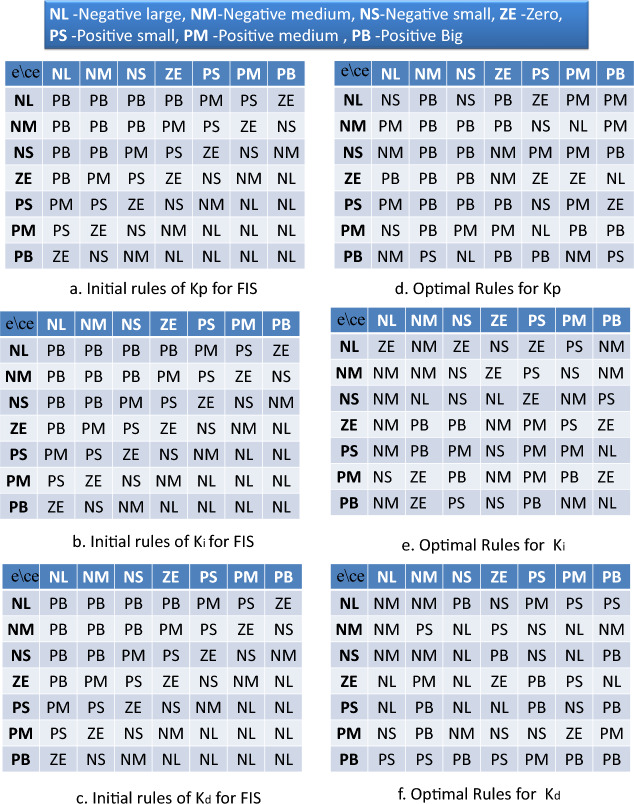
Rule base for fuzzy inference system. (**a**) Rules for ${{k}_{p}}$ of FIS. (**b**) Rules for ${{k}_{i}}$ of FIS. (**c**) Rules for ${{k}_{d}}$ of FIS. (**d**) Optimal rules for ${{k}_{p}}$ of FIS. (**e**) Optimal rules for ${{k}_{i}}$ of FIS. (**f**) Optimal rules for ${{k}_{d}}$ of FIS.

**Rule base for FIS** For the FIS system, a set of 49 rules for each of the output variables of the FIS is randomly set with knowledge of the performance of the PID controller for the system. The initial rules for the output variable ${{k}_{p}}$ of FIS are presented in Fig. 5a. Similarly, the rules for ${{k}_{i}}$ of FIS are presented in Fig. 5b. Rules for the ${{k}_{d}}$ of FIS are presented in Fig. 5c.

To optimize fuzzy rules, all linguistic rules must be converted to integer values so that one can easily generate the required number of randomised rules in numbers. Therefore, all linguistic rules are first normalized. A population of such normalized rules will be optimized through a metaheuristic algorithm. For this purpose, the following steps are continued in the proposed method.

**Step 2: Generation of a population of normalized initial fuzzy rules**

**Initialize stage:** The membership functions of inputs and outputs of the FIS are represented by integer values as follows:

15 $$\begin{aligned} \left[ \text {NL, NM, NS, ZE, PS, PM, PB} \right] =\left[ \text {0, 1, 2, 3, 4, 5, 6} \right] , \end{aligned}$$

where “0” represents NL (negative large), “6” represents PB (positive big) and so on.

**Initial generation of rules:** Initially, generate 49 random integer numbers within the range of 0 to 7 for each of the fuzzy output variables (${K_p}$, ${K_i}$ and ${K_d}$). Each number will be truncated to the next lowest integer. For example, let a randomly generated number within [0,7] is 1.4. It will be truncated to 1; 0.25 will be 0, 3.6 will be 3, etc. Such normalized integer numbers will be assigned as rules for the FIS, which means the number 1 represents NM (negative medium) or 0 is NL (negative large), etc. Mathematically, the above steps are described as follows:

The optimization variables are as follows:

16 $$\begin{aligned} X=\left[ \begin{matrix} {{X}_{{{k}_{p}}}} &{} {{X}_{{{k}_{i}}}} &{} {{X}_{{{k}_{d}}}} \\ \end{matrix} \right] , \end{aligned}$$

where $${{{X}_{{{k}_{p}}}}=\underbrace{\left[ \begin{matrix} {{x}_{{{k}_{p1}}}} &{} {{x}_{{{k}_{p2}}}} &{} \cdots &{} \cdots &{} {{x}_{{{k}_{p49}}}} \\ \end{matrix} \right] ^T}_{\text {49 rules for }{{k}_{p}}}}$$; $${{{X}_{{{k}_{i}}}}=\underbrace{\left[ \begin{matrix} {{x}_{{{k}_{i1}}}} &{} {{x}_{{{k}_{i2}}}} &{} \cdots &{} \cdots &{} {{x}_{{{k}_{i49}}}} \\ \end{matrix} \right] ^T}_{\text {49 rules for }{{k}_{i}}}}$$; and

$${{{X}_{{{k}_{d}}}}=\underbrace{\left[ \begin{matrix} {{x}_{{{k}_{d1}}}} &{} {{x}_{{{k}_{d2}}}} &{} \cdots &{} \cdots &{} {{x}_{{{k}_{d49}}}} \\ \end{matrix} \right] ^T}_{\text {49 rules for }{{k}_{d}}}}$$.

For *N* number of population, the initial generation of rules in the terms of integer number for each fuzzy output variable will be as follows:

17 $$\begin{aligned} {{X}_{N}}={{\left[ \begin{matrix} {{X}_{{{k}_{p1}}}} &{} {{X}_{{{k}_{i1}}}} &{} {{X}_{{{k}_{d1}}}} \\ \vdots &{} \vdots &{} \vdots \\ {{X}_{{{k}_{pN}}}} &{} {{X}_{{{k}_{iN}}}} &{} {{X}_{{{k}_{dN}}}} \\ \end{matrix} \right] }_{ 49\times N \times 3}}. \end{aligned}$$

where *N* is number of population.

Randomly generated normalized rules are required to convert into linguistic terms for use in the FIS of the Fuzzy-PID controller.

**Step 3: Set new rules in FIS using randomly generated normalized rules- conversion of normalized rule to linguistic form**

The randomly generated population of normalized rules will be converted to text form to update rules in FIS for the Fuzzy-PID controller as follows:

- **Step 3.1: Get “ if” portion of fuzzy rules-** The antecedents, or “if” portion, for each rule index are directly taken from the “if” portion of the rule base FIS as designed in ’Step 1’.
- **Step 3.2: Create “then” portion of fuzzy rules-** For each fuzzy output variable index, the consequents or “then” portions of fuzzy rules are created from the normalized rules.
- **Step 3.3: Set rules as text-** For degree of support 1, the fuzzy rules will be set on the basis of antecedents, consequents, and FIS for each rule index.

**Step 4: Defuzzification method**

By using the defuzzification method, the fuzzy values of the designed variables are transformed into exact values, which are then provided to the PID controller for tuning. The defuzzification approach utilised in this paper is the area barycenter method.

Step 3 and 4 will be repeated at each iteration until the termination criterion (maximum number of iterations) is met. An optimization algorithm will be used for this purpose. For a fixed number of iterations, the normalized rules will be updated, and the best one will be selected for the ventilator system.

**Step 5: Optimizing FIS rules**

The fuzzy rules, as generated randomly in Step 3, will be optimized by using a metaheuristic algorithm. For this purpose, a reshaped class topper optimisation algorithm has been developed. The details of the algorithm are presented in the next section. The fitness function will be evaluated each time to identify the best rules for FIS by the RCTO. The following performance index will be assessed to reduce the discrepancy between the deserved airway pressure and the pressure of the ventilator system.

**Fitness function**

The fitness function in the ventilation system is considered as a integral-time absolute error (ITAE) among the intended airway pressure and the actual airway pressure. It is defined as follows:

18 $$\begin{aligned} {\Im }_1=\int \limits _{0}^{T}{t{|e|}dt}, \end{aligned}$$

where $${{E_1}={P_{airway}(desired)-{P_{airway}}(actual)}}$$.

Subject to the following constraints:

- $${0<{{X}_{{{k}_{p}}}}<7}$$;
- $${0<{{X}_{{{k}_{i}}}}<7}$$;
- $${0<{{X}_{{{k}_{d}}}}<7}$$.

where the term ${{X}_{{{k}_{p}}}}$, ${{X}_{{{k}_{i}}}}$ and ${{X}_{{{k}_{d}}}}$ are membership functions for output variables (${{k}_{p}}$, ${{k}_{i}}$, ${{k}_{d}}$) of the fuzzy controller. As stated in (15), a truncated number generated between o to 7 will represent a MF.

## Reshaped class topper optimization algorithm-RCTO

### Basic class topper optimization algorithm

A meta-heuristics optimization approach called class topping optimization (CTO) has been proposed in^31^. The idea of the CTO is based on the learning strategy of a student in a class to become the best in the class. According to the CTO, there must be a topper (section topper) in each section. The best one among all the section toppers is identified as the class topper. Section toppers improve their performance by learning from the class topper. Other students in each section will improve their knowledge by learning from the corresponding section toppers. All participants will therefore, continuously improve their skills at each stage of the tests. The advantage of this algorithm is that, the complete search space of an optimization problem is divided into small sections (local search spaces). In each examination or iteration, the toppers (local solutions) in each section (local search space) are found during the exploitation stage of the CTO algorithm. Then, among all the local toppers, the best one (class topper or global solution) is selected. During the exploitation stage of CTO, each local solution (section toppers) will be updated on the basis of the class topper (global solution) and other solutions (students) will be updated based on section toppers.

Let’s assume that a class has (*SEC*) sections and (*L*) pupils in each section. The test will be repeated (*j*) times. The CTO has upgraded learner performance (*Prf*) as follows::

### Updation rules for section topper (local optimum)

19 $$\begin{aligned} {Ex^{ST}}_{(L,j+1)}=Whf_{t_1}*{Ex^{ST}}_{(L,j)}+{Ca_1}*rd_{1}*(CT_{j}-ST_{j}), \end{aligned}$$

20 $$\begin{aligned} {Prf^{ST}}_{(L,j+1)}={Prf^{ST}}_{(L,j)}+{Ex^{ST}}_{(L,j+1)}, \end{aligned}$$

where ${{Ex^{ST}}_{(L,j+1)}}$ represent the grades of ${L^{th}}$ pupil at ${(j+1)^{th}}$ test, ${Whf_{t_1}}$ is weight scale, ${Ca_1}$ is acceleration factor, $rd_{1}$ is random gain, ${{Ex^{ST}}_{(L,j)}}$ grades of ${L^{th}}$ pupil or search agent at ${j^{th}}$ test, ${CT_{j}}$ represents the class-leader at ${j^{th}}$ test, ${ST_{j}}$ is the leader in a section at ${j^{th}}$ test, ${Prf^{ST}}_{(L,j+1)}$ represent the updated achievement of ${L}^{th}$ pupil in the ${(j+1)^{th}}$ test, ${Prf^{ST}}_{(L,j)}$ is the score of ${L^{the}}$ pupil at ${j^{th}}$ test.

### Updation rules of students (search agent) in a section (local space)

21 $$\begin{aligned} {Ex^{S}}_{(L^{'},j+1)}=Whf_{t_2}*{Ex^{S}}_{(L^{'},j)}+{Ca_2}*rd_{2}*(ST_{j}-{L^{'}}_{j}), \end{aligned}$$

22 $$\begin{aligned} {Prf^{S}}_{(L^{'},j+1)}={Prf^{S}}_{(L^{'},j)}+{Ex^{S}}_{(L^{'},j+1)}, \end{aligned}$$

where ${Ex^{S}}_{(L^{'},j+1)}$ is marks of ${L^{'}}^{th}$ learner in the ${(j+1)^{th}}$ examination, ${Whf_{t_2}}$ is weight-factor, ${{Ca_2}}$ is cofactor of acceleration, ${rd}_{2}$ is random value, ${{Ex^{S}}_{(L',j)}}$ is score of ${L'}$ learner in the ${j^{th}}$ test. ${ST_{j}}$ is the section-topper at ${j^{th}}$ test, ${L^{'}}_{j}$ is the grade in the ${j^{th}}$ test $({L^{'}}_{j}\ne {CT_{j}}\ne {ST_{j}})$, ${Prf^{S}}_{(L^{'},j+1)}$ is the performance measured for ${L'}$ learner at ${(j+1)^{th}}$ test, ${Prf^{S}}_{(L^{'},j)}$ is the achievement of ${L'}$ learner at ${j^{th}}$ test.

### Proposed RCTO for optimizing fuzzy rules

**Figure 6:**
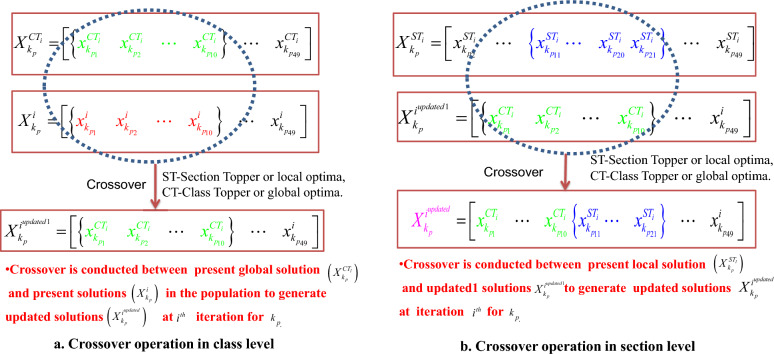
Crossover operations for RCTO. (**a**) Crossover in class level. (**b**) Crossover in section level.

In CTO, the complete search space (class) is divided into local search spaces (sections). The algorithm starts to find better solutions in each local search space. Then, the best one among all local optimal solutions (section toppers) is identified as the global solution (class topper) in the search space (class). According to the CTO, all local solutions (section toppers) in an iteration will be updated on the basis of the global solution in the same iteration. In RCTO, the local and global solutions in a particular iteration will be identified according to the classical CTO. The updating stage of initial solutions as presented in (19) to (22) will be reshaped to form RCTO for optimizing normalised fuzzy rules for FIS to design the Fuzzy-PID controller for ventilator models. The concept of reshaping is taken from the concept of crossover in evolutionary algorithms. The performance of the Fuzzy-PID controller with the initial rule base will be evaluated for the ventilator model (9) with the fitness function (18) at each iteration. According to the fitness values, a set of current best rules in each section (section toppers or local solutions) is identified. The best set of rules among all section toppers (local solutions) are also identified as the global best solution or class topper. Then, the cross-over technique is applied in two ways to generate new, updated solutions for the next iteration, as follows:

- *Crossover in class level* In this stage, each randomly generated normalized rule for each of the fuzzy output variables will be updated on the basis of the current global solution or class topper (CT). The crossover operation will be conducted among each randomly generated normalized rule and the current best solution at an iteration at randomly selected positions in the search space. For this purpose, any number of rules of a fuzzy output variable from the current best solution among all local solutions (STs) are selected as CT. In this section, 10 rules for each fuzzy variable from the current best solutions are selected. One may follow different numbers of rules according to the chosen number of MF for a variable. For the same position in the present solutions, the crossover technique is applied. For ease of presentation, this step is presented below for a single variable. For other fuzzy output variables, the crossover technique will be the same. Let a set of randomly generated rules for fuzzy output variable $k_p$ is as follows:
  23 $$\begin{aligned} {{{X}_{{{k}_{p}}}}=\underbrace{\left[ \begin{matrix} {{x}_{{{k}_{p1}}}} &{} {{x}_{{{k}_{p2}}}} &{} \cdots &{} {{x}_{{{k}_{p10}}}} &{} \cdots &{} {{x}_{{{k}_{p49}}}} \\ \end{matrix} \right] ^T}_{\text {49 rules for }{{k}_{p}}}} \end{aligned}$$
  The crossover steps for fuzzy output variable ${k_p}$ is shown in Fig. 6a. In the diagram, for simplicity, the first 10 rules of the variable are shown to participate in the crossover technique. The rules can be selected randomly with any number. For other fuzzy output variables ($k_i$, and $k_d$), the crossover will be done in the same way.
- *Crossover in section level* In this stage, each updated rule on the basis of a class topper (CT) or global solution will again participate in a crossover operation with the section toppers (STs) or local solutions in each section (local search space). The updated rules of a variable in the previous stage will be merged with the current best solution in a section (section topper or local optima) at the same iteration at a randomly selected position. For this purpose, any number of fuzzy output variables from the best solutions (section toppers, or ST) in each section are selected. In this paper, 10 randomly selected rules for each fuzzy variable form the current best solutions. One may follow different numbers of rules. For the same position in the present updated solutions, the crossover technique is applied. For the fuzzy output variable ${k_p}$, the updation is shown in Fig. 6b. In the diagram, for simplicity, 10 rules between rules 11 and 20 of the variable ${k_p}$ have been presented to show the crossover technique. The rules can be selected randomly with any number. For other fuzzy output variables (${k_i}$, and ${k_d}$), the crossover will be done in a similar way. After reaching the termination criterion, the best set of rules will be generated for each of the output variables of FIS for the Fuzzy-PID controller for the ventilator model.

## Stability analysis

In this section, the stability of the closed-loop ventilator system based on the Fuzzy-PID controller with RCTO optimization is analyzed. The stability of the system is checked using the Nyquist determination approach. The transfer function of the closed-loop system is required to evaluate stability. The closed-loop transfer function of the optimized Fuzzy-PID-controlled ventilator is presented using a bilinear transformation in (27) with the transfer function of the ventilator model $(G\left( s \right) )$ (10), blower model $({G}_{blower}\left( s \right) )$ (11), and the proposed controller as equivalent to ${{G_{PID}(s)} =\frac{{k_p}s + {k_i}+{k_d}{s^2}}{s}}$.

The transfer function of the ventilator model (10) is presented as follows:

24 $$\begin{aligned} G\left( s \right) =\frac{0.5063 s + 5.063}{1 s + 5.443} \end{aligned}$$

The transfer function of the blower model (11) is presented as follows:

25 $$\begin{aligned} {G}_{blower}\left( s \right) =\frac{35530}{s^2 + 377 s + 35530} \end{aligned}$$

The transfer function of the PID controller using the optimal parameters set by the optimal rule based fuzzy controller optimized with RCTO, as presented in Table 2, is presented as follows:

26 $$\begin{aligned} {G_{PID}(s)}=\frac{ 0.00048 s^2 + 0.2891 s + 62.88}{s} \end{aligned}$$

The closed-loop transfer function of the proposed control scheme for the ventilator model is found as follows:

27 $$\begin{aligned} \frac{y(s)}{r(s)}=\frac{8.635 s^3 + 5287 s^2 + 1.183e06 s + 1.131e07}{s^4 + 391.1 s^3 + 4.287e04 s^2 + 1.377e06 s + 1.131e07} \end{aligned}$$

Consider ${{G_{PID}(s)}{G\left( s \right) {{G}_{blower}\left( s \right) }}}$ as the open-loop transfer function of the system. The open-loop Nyquist curve of the system (${{G_{PI}(j\omega )}{G\left( j\omega \right) {{G}_{blower}\left( j \omega \right) }}}$) must not include point $(-1,j0)$ in order for the closed-loop system to be stable when ${\omega }$ vary from 0 (zero) to infinity^32^.

**Figure 7:**
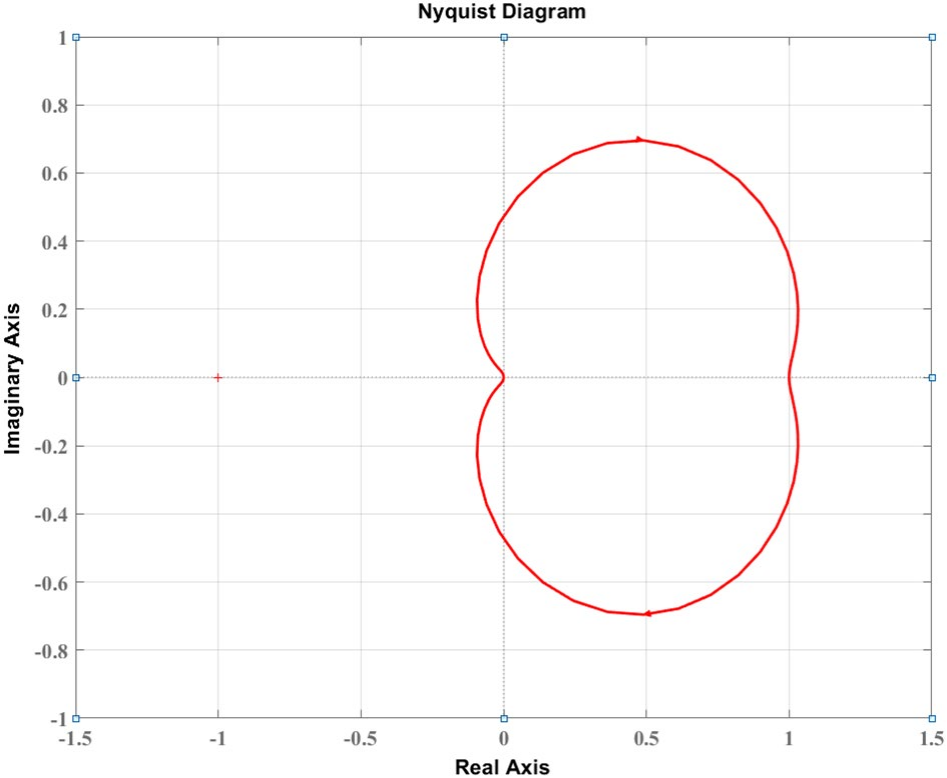
Nyquist diagram of the pressure control for the ventilator based on optimal ${Fuzzy-PID}$.

The Nyquist plot of the pressure control system for ventilator model based on Fuzzy-PID-RCTO is produced in MATLAB and illustrated in Fig. 7. It is observed in Fig. 7 that the system does not include the $(-1,j0)$ point. Thus, the Fuzzy-PID-RCTO-based ventilator pressure control system is closed-loop stable.

## Simulation and result analysis

The simulation of the proposed control strategy for the ventilator model is presented in this section. For a set breathing profile, the rules of the FIS of the Fuzzy-PID controller are first optimized with the RCTO algorithm. Then, with the optimized rule-based FIS, the Fuzzy-PID controller is examined for the ventilator model under different scenarios.

**Figure 8:**
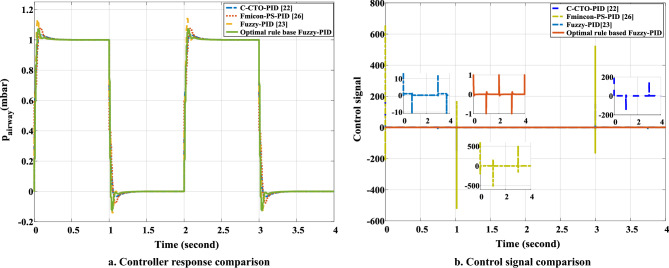
Controller performance. (**a**) Comparison of pressure tracking response of ventilator. (**b**) Comparison of control signal generated during simulation.

The transfer function model of the ventilator, the proposed controller, and the RCTO algorithm are all implemented on the LabVIEW©2015 platform. The values of the parameters of the ventilator model are presented in Table 1. A unit pulse signal with an initial value of 0, a final value of 1, and a period of 2 is set as the desired pressure profile for the ventilator system. The simulation is done for 4 seconds. In this scenario, the RCTO algorithm is run for the ventilator model with the proposed control structure (Fig. 2) for the fitness function as described in (18). After a fixed number of iterations as a termination criterion (Table 1), the optimal set of rules of fuzzy variables (membership functions of ${{k}_{p}}$, ${{k}_{i}}$, and ${{k}_{d}}$) are obtained for the ventilator model as presented in Fig. 5d,e, and f, respectively. In Fig. 5, it is observed that most of the randomly selected rules for fuzzy output variables (${{k}_{p}}$, ${{k}_{i}}$, and ${{k}_{d}}$) are changed with optimal settings. In Fig. 5a, when error and change in error are both negative large (NL), the rule for ${{k}_{p}}$ is randomly set as positive big (PB). After optimizing rules for ${{k}_{p}}$, an optimal rule of negative small (NS) is found instead of a randomly selected MF of positive big (PB), as shown in Fig. 5d. Similarly, most of the other rules for fuzzy output variables are found to be changed after optimizing them with RCTO, as shown in Fig. 5d,e, and f respectively. With membership functions of fuzzy variables (Figs. 3 and 4) and the obtained optimal rules (Fig. 5d,e, and f), the proposed Fuzzy-PID controller is verified for the ventilator for the same breathing pattern of the unit pulse signal with initial value 0, final value 1, and period 2. The gains of the PID controller set by the optimal FIS are found as follows: ${{k}_{p}}$ is 0.289085, ${{k}_{i}}$ is 62.8799, and ${{k}_{d}}$ is 0.000480583. The pressure-tracking efficiency of the ventilator with the optimal rule-base Fuzzy-PID controller is illustrated in Fig. 8a. It is seen in Fig. 8a that the pressure pattern of the ventilator system is effectively monitoring the intended pressure. A significant improvement in the pressure traceability profile has been observed in terms of peak time (${{t}_{p}}(s)$), overshoot (${{M}_{p}}$), and settling time (${{t}_{s}}(s)$) as tabulated in Table 2. It can be observed in Fig. 8a that the overshoot in pressure profile is improved by 16% in comparison with Fuzzy-PID where rules are randomly selected^30^. The overshoot in the pressure profile shown in Fig. 8a, is also low as compared to other methods for the same system. Settling time and peak time are also improved 60–80% compared to the existing method. The control signal for such a case is shown in Fig. 8b. The fuzziness of the proposed controller causes the control signal to be unsteady for a very brief period of time. Additionally, it has been found that the controlling signal obtained using the optimal rule based Fuzzy-PID has a substantially lower amplitude than the results obtained so far^16^. Higher magnitude actuating signals necessitate larger actuators, which may create an electric risk during the ventilation cycle. A massive motor is also expensive. Control signal generated by the proposed controller is also improved in magnitude by 80% to 90% compared to the existing method. Therefore, a modest motor is sufficient to get the ventilator to work as required with the proposed controller^33^.

**Table 2:** Performance comparison of optimal rule base Fuzzy-PID for respiratory ventilator system.

Method	Controller parameter			Controller performance		
	${{K}_{p}}$	${{K}_{i}}$	${{K}_{d}}$	${{M}_{p}}(\times {{10}^{-4}})$	${{t}_{p}}(s)$	${{t}_{s}}(s)$
Fmincon-PS based PID^33^	1.62	131.9539	0.025	1.12	0.005	0.18
C-CTO-PID^16^	1.65	131.3	0.006	1.02	0.005	0.06
Fuzzy-PID^30^	0.77034	217.7555	0.0125	1.7	0.005	0.06
Optimal rule based Fuzz-PID	0.289085	62.8799	0.000480583	1.5	0.001	0.002

**Figure 9:**
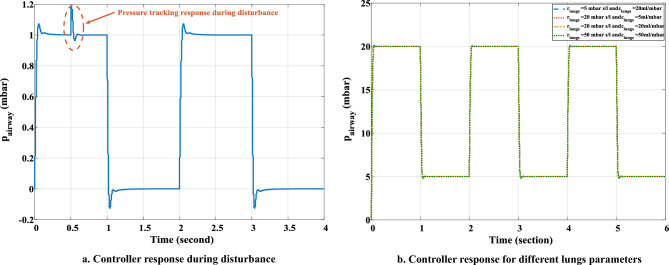
Proposed controller response. (**a**) Controller performance under external disturbance in pressure profile. (**b**) Controller performance under different lung conditions.

An airway pressure disruption of ${1\%}$ for 0.5 s is introduced in order to verify the response of the optimized Fuzzy-PID controller for the ventilator system under external disturbance. Figure 9a shows how the system performed while there was a perturbation. The turbulence is effectively solved by the suggested controller, and the pressure pattern gradually approached the ideal profile. The reliability of the suggested controller is also examined for variations in lung parameters such as compliance and resistance. For this purpose, a baseline respiration cycle of 5 mbar of PEEP and 20 mbar of PIP with a desired cycle time of 2 s is considered. In this case, the action of the controller is observed and illustrated in Fig. 9b. The suggested control strategy worked effectively to settle the airway pressure profile to the desired profile under such parametric changes.

**Figure 10:**
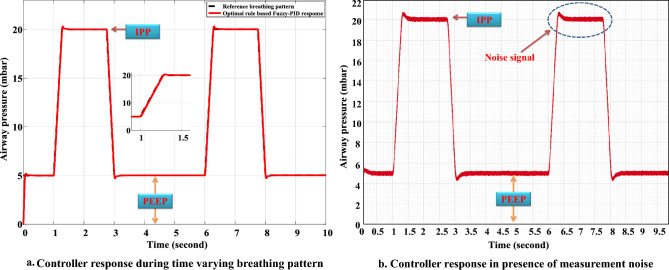
Proposed controller response. (**a**) Controller performance under time varying breathing pattern. (**b**) Controller performance under time varying breathing pattern in presence of measurement noise.

The ideal rhythm for breathing may alter over time according to the patient’s labour during ventilation. Thus, to verify the resilience of the suggested controller, a variable breathing pattern of 5 mbar of PEEP, 20 mbar of PIP, 2 s of inspiration time, and 3 s of expiration time is also examined. In this scenario, the gains of the PID controller tuned by the optimal rule-based FIS are obtained as ${{k}_{p}}$ is 0.861544, ${{k}_{i}}$ is 215.268 and ${{k}_{d}}$ is 0.0119251 for the ventilator model. The performance of the controller is also illustrated in Fig. 10a. It can be observed that the pressure profile of the ventilator is tracking the reference breathing pattern with a considerable amount of overshoot for a short duration of time. To examine the response of the controller in the presence of measurement noise, the system is assumed to have a gaussian white noise of zero mean with 1${\%}$ standard deviation. It is demonstrated how the ventilator reacts to such measurement noise in Fig. 10b.

### Remark 1

During optimizing the fuzzy rules of the proposed Fuzzy-PID controller with the RCTO algorithm for the ventilator model, the RCTO algorithm takes 158.12 s when run on an Intel (R), Core(TM) ${i7-8700}$, 3.20–3.19 GHz CPU, and 32.0 GB of RAM desktop computer. It is not necessary to continuously optimise the rules of the proposed controller under other circumstances once they have been optimised for the ventilator system. Under any disturbance, a fuzzy controller can change the gains of the PID controller for the ventilator model. The ventilator model’s pressure is observed to be controlled by the optimised Fuzzy-PI controller in 0.013 s when there is a disturbance of 1${\%}$change in airway pressure at 0.5 s (Fig. 9a). It takes 0.163 s to adjust the PID controller to achieve the required response for a breathing pattern that varies over time (Fig. 10).

## Conclusion and future work

The aim of this paper is to design an efficient Fuzzy-PID controller to enhance the pressure monitoring performance of an artificial ventilator. Therefore, a systematic method is developed to optimize the rules of the fuzzy inference system of the suggested control structure. The designed controller is validated with a blower-driven patient-hose ventilator model operated in pressure-controlled ventilation mode. The simulation results show the effectiveness of the control scheme in different scenarios. The proposed controller is able to adjust the PID gains under parametric uncertainties and disturbances. In comparison to previous findings, the ventilator model’s ability to track pressure is enhanced in terms of response time, overshoot, and steady-state error.

The proposed method is simple and effective for designing a robust, optimal rule-based fuzzy controller for ventilation models. The accuracy of the proposed scheme can be further improved by using a fractional-order membership function. One of the drawbacks of the optimizing FIS is that during the periods of optimizing rules, the MFs will try to fix themselves, which may change the response of the system in real-world applications. One may extend the present work to overcome such an issue. The computation time of the fuzzy controller can also be taken care of in future work. In this paper, the linear patient-hose blower driven ventilator model is examined with the proposed control scheme. As biological systems are complex and non-linear in nature. Thus, optimizing such a large number of fuzzy rules with a swarm based optimization algorithm may not provide an optimal solution for a nonlinear model of a ventilator. In this regard, one may verify a reinforcement learning-based Q-learning optimization technique^34^ for the nonlinear ventilator model as a future scope in this field.

## Data Availability

The corresponding author will disclose the datasets utilized and/or processed throughout the current work upon reasonable request.
